# Dietary Intake Levels of Iron, Copper, Zinc, and Manganese in Relation to Cognitive Function: A Cross-Sectional Study

**DOI:** 10.3390/nu15030704

**Published:** 2023-01-30

**Authors:** Dong Zhao, Yilun Huang, Binghan Wang, Hui Chen, Wenfei Pan, Min Yang, Zhidan Xia, Ronghua Zhang, Changzheng Yuan

**Affiliations:** 1 Department of Nutrition and Food Safety, Zhejiang Provincial Center for Disease Control and Prevention, Hangzhou 310051, China; 2 School of Public Health, Zhejiang University School of Medicine, Hangzhou 310051, China; 3 College of Biosystem Engineering and Food Science, Zhejiang University, Hangzhou 310051, China

**Keywords:** diet, trace metal, cognitive function, cross-sectional study

## Abstract

**Background**: Previous studies have related circulating levels of trace metal elements, of which dietary intake is the major source, to cognitive outcomes. However, there are still relatively few studies evaluating the associations of dietary intake levels of iron, copper, zinc, and manganese with cognitive function (CF). **Methods**: We leveraged the data of 6863 participants (mean [standard deviation] age = 66.7 [10.5] years) in the Health and Retirement Study (2013/2014). Dietary intake levels of iron, copper, zinc, and manganese were calculated from a semi-quantitative food frequency questionnaire. CF was assessed using the 27-point modified Telephone Interview for Cognitive Status (TICS). We used linear regression models to calculate the mean differences in global CF scores by quintiles of dietary intake levels of trace metal elements. **Results**: Among the study participants, the mean (SD) values of daily dietary intake were 13.3 (6.3) mg for iron, 1.4 (0.7) mg for copper, 10.7 (4.6) mg for zinc, and 3.3 (1.6) mg for manganese. Compared with the lowest quintile of dietary iron intake (<8.1 mg), the highest quintile (≥17.7 mg) was associated with a lower cognitive score (−0.50, −0.94 to −0.06, P-trend = 0.007). Higher dietary copper was significantly associated with poorer CF (P-trend = 0.002), and the mean difference in cognitive score between extreme quintiles (≥1.8 vs. <0.8 mg) was −0.52 (95% confidence interval: −0.94 to −0.10) points. We did not observe significant associations for dietary intake of zinc (P-trend = 0.785) and manganese (P-trend = 0.368). **Conclusion**: In this cross-sectional study, higher dietary intake of iron and copper was related to worse CF, but zinc and manganese intake levels were not significantly associated with CF.

## 1. Introduction

In the context of global aging [1,2], cognitive impairment and dementia, regarded as the major causes of disability and mortality in older adults, place a great burden on healthcare systems and resources [3,4,5]. The lack of effective treatments for dementia [6,7,8] makes it important to identify its risk factors for primary prevention [9,10].

Trace mineral elements, including iron, copper, zinc, and manganese, could be associated with brain health through several biological pathways [11,12,13]. Zinc is an important component of various enzymes in the nervous system [14,15], while iron, copper, and manganese could participate in the process of inflammatory, oxidative, and stress response of neurons [16,17,18]. Previous epidemiologic research has also revealed that higher serum iron and copper, and lower serum zinc and manganese may be associated with worse cognitive function [19,20,21,22].

However, apart from the existing evidence from animal studies, [23,24,25] population-level evidence of dietary intake levels of these elements with cognitive outcomes is still limited and inconsistent [26,27,28]. In most previous studies, dietary iron, mainly from red meat, nuts, and dark green leafy vegetables [27], was negatively associated with cognitive function [27,29]. For example, higher iron intake was associated with a 54% higher risk of mild cognitive impairment in an Australian cohort [27]. In terms of dietary copper, which is mainly from nuts and seafood [30], a previous study found that a higher copper intake, particularly when combined with a diet high in saturated fat, was associated with a higher risk of dementia and cognitive impairment [31], while another study reported the opposite [28]. In addition, most previous population studies on the associations of dietary zinc and manganese with cognitive function were conducted among children [32,33], while evidence for age-related cognitive function among older adults remained scarce [28,34].

Hence, we aimed to explore the relations between dietary intake levels of iron, zinc, manganese, copper, and CF from the Health and Retirement Study (HRS), a nationally representative population-based study in the U.S.

## 2. Materials and Methods

### 2.1. Participants

The current study was a cross-sectional analysis based on the core 2014 survey of the Health and Retirement Study (HRS) and the Health Care and Nutrition Study (HCNS). Details of the HRS have been described elsewhere [35]. Briefly, the HRS is a national longitudinal study among people over 50 years old in the United States. Participants have been revisited biennially for their health and financial situation since 1992 and for cognitive function since 1996. Proxy respondents were sought for people who were unwilling or unable to finish an interview themselves (~9%). The response rate of HRS was up to 85% [36]. The HCNS conducted in 2013–2014, with a 65% sample response rate, was a sub-study of the HRS. It provided measurements of health care access, food purchases, food consumption, and nutrition (including vitamins and other supplements).

The Health and Retirement Study has been approved by the Health Sciences and Behavioral Sciences institutional review board at the University of Michigan (IRB Protocol: HUM00061128). Written informed consent to participate in the HRS was obtained from all respondents or proxies.

In the present study, we included 6863 participants (Figure S1) from the HCNS who: (1) completed the cognitive test; (2) were not less than 50 years old when finishing the HCNS; (3) reported reasonable energy intake (600–4200 kcal/d for men and 500–3600 kcal/d for women) [37].

### 2.2. Measurements of Dietary Intake of Iron, Zinc, Manganese, and Copper

Dietary intake levels were measured utilizing the validated 163-item semi-quantitative Harvard Food Frequency Questionnaire (FFQ) [38], which has been validated against multiple 24-h recalls and relevant biochemical indicators. Its applicability has been confirmed by other published studies using the HRS [39,40]. In this study, we took dietary intake levels of iron (Fe), copper (Cu), zinc (Zn), and manganese (Mn) (their correlations were shown in Table S1) as exposures. The intake of each food group (not including dietary supplements) was converted from categorical responses to numeric variables, and the total nutrient intake was calculated with a food composition table [41]. The continuous intake levels were categorized into quintiles according to the population distribution.

### 2.3. Cognitive Function

In this study, the primary outcome was cognitive function, measured using the adapted Telephone Interview for Cognitive Status (TICS) on a 27-point scale [42]. The test included an immediate and a delayed 10-noun free recall test (1 point for each, 20 points in total), backward counting from 20 (2 points for correct on the first try, 1 point for correct on the second try, 2 points in total), and serial 7 subtractions (i.e., subtract 7 from 100 and continue subtracting 7 from each subsequent number for a total of five trials, 1 point for each trial, 5 points in total). Our secondary outcome was cognitive impairment, which was defined as less than 12 points in the TICS [42].

### 2.4. Covariates

We used directed acyclic graphs to identify the possible confounders of the relations of interest. Sociodemographic characteristics included age (year), squared age (year^2^), gender defined by self-identity, race (White or Caucasian/Black/Others), education (measured as years of formal education), household income (<20,000/20,000~40,000/40,000~80,000/>80,000, US $/year), and marital status (married or partnered/other situations). Lifestyle factors included smoking (current/former/never), alcohol drinking (current/former/never), vigorous exercise (never/>once per month/>once per week), and body mass index (BMI) categories (underweight/normal weight/overweight/obesity). Other dietary factors included total energy intake (kcal/d), vitamin supplementation intake (yes/no), iron supplementation intake (yes/no), zinc supplementation intake (yes/no), fat intake (g/day), and fiber intake (g/day). Moreover, health conditions included hypertension (yes/no, self or proxy reported), diabetes mellitus (yes/no, self or proxy reported), heart problems (yes/no, self or proxy reported), and depression (yes/no, by the validated 8-item Center for Epidemiologic Studies-Depression [CES-D] scale [43]).

### 2.5. Statistical Analysis

We described the baseline characteristics of the study population by gender, presenting the mean and standard deviation (SD) for continuous variables, and the number and percentage for categorical variables.

In the primary analysis, linear regression models were used to estimate β-coefficients and 95% confidence intervals (CIs) for the associations of dietary intake levels of trace metal elements with the global cognitive scores. Potential non-linear associations were examined by restricted cubic spline regression models with four knots at the 20th, 40th, 60th, and 80th percentiles. The median values of dietary intake levels of trace metal elements were treated as the reference points. Model 1 was adjusted for age, squared age, sex, and race. Model 2 was based on Model 1 and further adjusted for marriage status, education, physical activity, household income, smoking status, alcohol drinking status, and use of vitamin, iron, and zinc supplements. Based on Model 2, Model 3 was additionally adjusted for dietary intake of fat and fiber. We also conducted prespecified stratified analyses by age (<65/≥65 years), sex (Male/Female), cardiometabolic disease status (yes/no), BMI (<25/≥25 kg/m^2^), education (<10 years/10–15 years/≥15 years), marital status (married or partnered/other situations) [27], iron supplement intake (yes/no), and zinc supplement intake (yes/no).

In the secondary analysis, logistic models were used to examine the association between mineral intake levels and cognitive impairment (CI). Odds ratios (ORs) and 95% confidence intervals (CIs) were reported for quintiles of mineral intake levels, with the lowest quintile as the reference group.

To test the robustness of the main results, we conducted the following sensitivity analyses: (1) we further adjusted the models for hypertension, diabetes mellitus, heart problems, depression, BMI categories, and intake levels of pantothenic and phosphorous, sequentially; (2) we further excluded participants who had a history of stroke or dementia, respectively [27,44]; (3) to assess their independent associations, we mutually adjusted the models for other mineral intake levels; (4) we calculated the intake of minerals to total energy intake of 2500 kcal (Table S2) [45] to further account for the confounding effect of total energy intake [27].

All analyses were performed using R 4.1.2. Two-sided *p*-values and 95% confidence intervals (CIs) were reported throughout. The *p*-value < 0.05 represented statistical significance.

## 3. Results

### 3.1. Participants Characteristics

A total of 6863 participants were included, among whom 59.3% were female, 69.7% were White, 64.4% were married/partnered, and their mean (SD) age was 66.7 (10.5) years in 2014 (Table 1). The mean (SD) value of the participants’ global cognitive score was 15.3 (4.4) out of 27. The mean (SD) values of dietary mineral intake levels were 13.3 (6.3) mg for iron, 1.4 (0.7) mg for copper, 10.7 (4.6) mg for zinc, and 3.3 (1.6) mg for manganese (Table S2).

### 3.2. Associations of Mineral Intake Levels with Cognitive Function and Cognitive Impairment

Table 2 demonstrated the difference in the global cognitive function score among quintiles of mineral intake. In the fully adjusted model, iron (P-trend = 0.007) and copper (P-trend = 0.002) intake showed significant associations with CF. Compared to the lowest quintile of dietary iron intake (<8.1 mg), the second quintile (8.1–<10.8 mg) was related to a higher global cognitive score (beta = 0.30, 95% CI: 0.02 to 0.59), but the highest quintile (≥17.7 mg) was associated with a lower score (−0.50, −0.94 to −0.06). In contrast, higher copper intake was associated with poorer cognitive function in a dose-response manner, with the difference being −0.11 (−0.39 to 0.18) for quintile 2, −0.17 (−0.47 to 0.14) for quintile 3, −0.27 (−0.63 to 0.08) for quintile 4, −0.52 (−0.94 to −0.10) for quintile 5, compared to the lowest quintile. Higher zinc (P-trend = 0.003) and manganese (P-trend < 0.001) intake was related to better cognitive scores when adjusted for age, squared age, sex, and race, but the relations were non-significant in the fully adjusted models (P-trend = 0.785 for zinc and 0.368 for manganese). The associations were similar when we assessed the relations of the mineral intake to cognitive impairment (P-trend = 0.009 for iron, 0.018 for copper, 0.668 for zinc, and 0.944 for manganese, Table 3).

**Figure 1:**
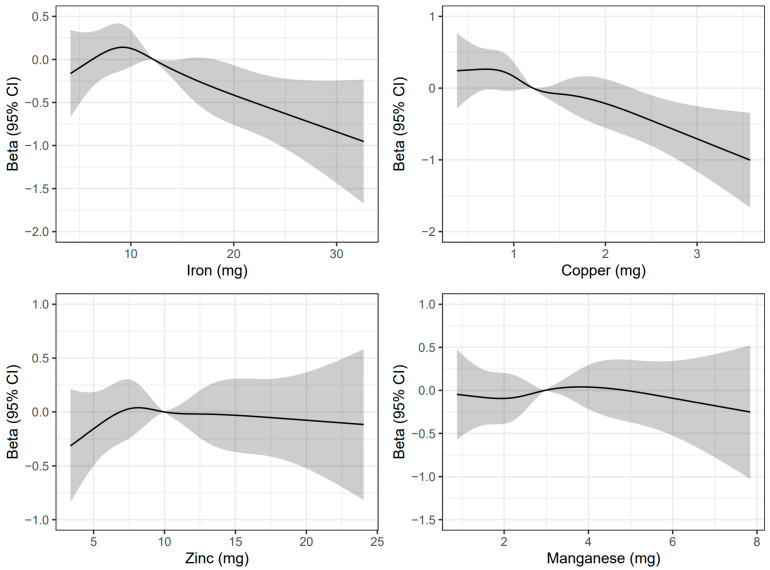
Restricted cubic splines for the association of dietary intake levels of iron, copper, zinc, and manganese with global cognitive score. The reference point was set as the median values of dietary intake levels of iron, copper, zinc, and manganese. P-nonlinear = 0.209 for dietary iron intake, 0.836 for dietary copper intake, 0.613 for dietary zinc intake, and 0.704 for dietary manganese intake. The solid line represents the beta of the linear model, and the shaded area represents the 95% CIs of beta.

The restricted cubic spline analysis confirmed and visualized the above-mentioned findings (Figure 1). While no significant nonlinear relations were observed (P-nonlinear = 0.209 for iron, 0.836 for copper, 0.613 for zinc, and 0.704 for manganese), an inverted J-shaped association was shown for dietary iron intake. In addition, higher dietary copper intake was related to poorer cognitive function (Figure S2). 

### 3.3. Subgroup Analyses

**Figure 2:**
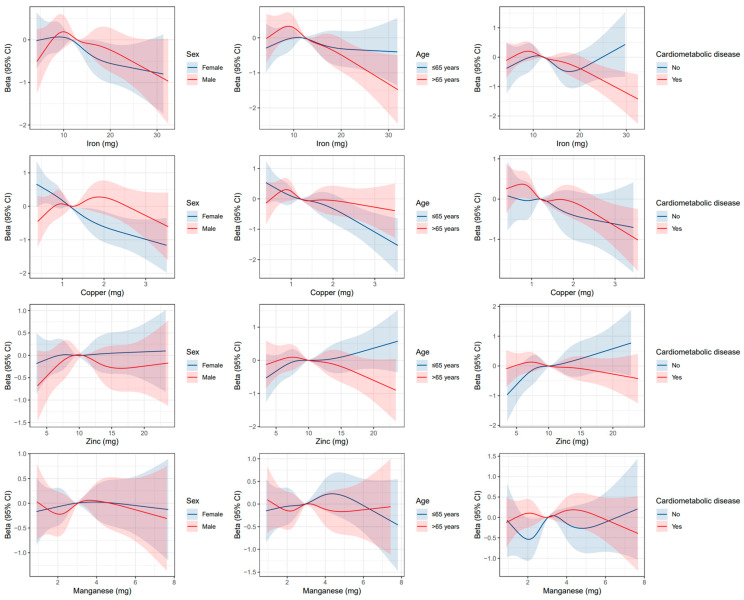
Restricted cubic splines for the association of dietary intake levels of iron, copper, zinc, and manganese with global cognitive score by age, sex, and cardiometabolic disease. The solid line represents the beta of the linear model, and the shaded area represents the 95% CIs of beta.

We conducted prespecified stratified analyses by age, sex, cardiometabolic disease status, body mass index, education, marital status, iron supplement intake, and zinc supplement intake (Table S3, Figure 2 and Figure S3). Moreover, the association of high dietary copper intake was stronger in female participants (P-interaction < 0.001) and participants with BMI ≥ 25 kg/m^2^ (P-interaction = 0.040). The detrimental association of higher iron intake was strengthened in those with zinc supplement intake (P-interaction = 0.027) or iron supplement intake (P-interaction = 0.016). The association of dietary zinc intake with CF was generally consistent in the stratified analyses. A significant association of higher dietary manganese intake with better CF was also detected in participants with BMI < 25 kg/m^2^ (P-trend = 0.033, P-interaction = 0.006). No other covariates significantly interacted with mineral intake in relation to CF.

### 3.4. Sensitivity Analyses

In the sensitivity analyses, the observed associations remained robust. Additional adjustments for health conditions did not substantially change the associations (Table S4). After excluding participants with low global cognitive scores or with a stroke history, the associations persisted (Table S5). Noteworthy, we observed significant associations for all four minerals of interest when further adjusting the other three mineral intake levels when treating certain mineral intake as exposure, but the associations remain generally consistent with the primary analysis (Table S5). Higher zinc and manganese intake was related to better CF (P-trend = 0.033 for zinc and 0.026 for manganese). The beta (95% CI) was 0.44 (−0.07 to 0.95) comparing the extreme quintiles of zinc, and 0.16 (−0.33 to 0.65) for manganese. The associations were also consistent with the primary analyses when we used standardized levels of mineral intake (Table S5).

## 4. Discussion

In this cross-sectional study, we found that higher iron and copper intake levels were related to poorer cognitive function. Non-significant associations of zinc or manganese with cognitive function were observed. The associations were generally consistent in the subgroup and sensitivity analyses.

The observed associations of higher dietary iron intake with worse CF were consistent with previous studies [27,46,47,48]. For instance, Shi et al. reported that higher dietary iron intake (median: 31.7, 23.7, and 19.3 vs. 14.6 mg/d) was significantly associated with poorer cognition in Chinese adults [29]. Notably, foods rich in iron, such as red meat and liver [27,46], have also been related to increased odds of cognitive impairment in later life [49] in the Singapore Chinese Health study. It is worth mentioning that higher dietary iron intake (≥17.7 mg/d at the top quintile) in our study was much larger than the Recommended Dietary Allowance (RDA) of 8 mg/d for men and postmenopausal women and approximately equal to the RDA of 18 mg/d for premenopausal women according to the Dietary Reference Intakes (DRIs) [50] in North America. Therefore, our findings support the potential detrimental role of excessive iron intake in cognitive function among adults. Additionally, we observed that the second quintile (8.1–10.8 mg/d) was related to better CF compared with the lowest quintile (<8.1 mg/d), which suggested that iron might be associated with CF in a non-linear pattern [51]. These findings could be explained by previous evidence that iron was associated with increased free radicals, which induces cellular oxidative stress and neurodegenerative processes [51,52], and high dietary iron could result in iron deposition [24] related to brain functions [53]. However, more studies are warranted, considering that multiple iron-rich food groups might be associated with better CF, and heme and non-heme iron may have different associations with CF [27,54,55].

Additionally consistent with most previous studies [31,48,56], our study found an inverse association between dietary copper intake and CF, with the level at the top quintile (≥1.8 mg/d) far above the corresponding RDA (0.9 mg/d for adult men and women) [50]. For instance, Morris et al. found that higher dietary copper intake was associated with accelerated cognitive decline among participants with a high intake level of saturated and trans fats [57]. The association might be explained by copper-induced oxidation and inflammation. Copper could combine with Aβ to produce Reactive Oxygen Species [58], or oxidize some fat molecules into derivatives toxic to neurons [16,17,59], and excess copper might lead to inflammation by increasing the ceruloplasmin leading to higher risks for some neurological diseases [60,61]. However, an opposite association has also been reported in a cross-sectional study [28] probably due to differences in assessment methods for cognition and dietary intake. Future long-term studies are needed to reveal the association between copper intake and the intake of foods rich in copper with cognitive outcomes.

In addition, we observed non-significant associations for zinc and manganese intake. In a previous cross-sectional study, the association of zinc intake and CF was non-linear and became non-significant when the intake level was above the inflection point (8.94 mg/d in males and 7.58 mg/d in females) [34], which was comparable with quintiles 2 to 5 in our study. However, given that many studies revealed a positive association between zinc intake and CF [28,62,63], their relationship, especially zinc intake at a lower level, needs further explanation. In this study, the manganese intake levels were mostly below the tolerable upper limit (11 mg/day) in the DRIs [50] in North America among most participants (99.9%), which might account for the non-significant association [64]. However, we should also note that manganese could come from foods rich in neuroprotective elements [65], which may somewhat conceal its potential effects.

The large and nationally representative sample and the use of the validated FFQ were advantages of the present study. However, there are several limitations that need to be taken into account when interpreting our results. First, as a cross-sectional study, the observed associations might not necessarily be causal because reverse causality might exist. The long-term associations of these dietary mineral intake levels with cognitive function are yet to be revealed. Second, dietary intake levels of the minerals of interest were measured by the single FFQ, and measurement errors cannot be avoided. Furthermore, although we made the most possible adjustments for potential confounding factors, residual confounders are inevitable in the observational setting. Specifically, as the nutrients are highly correlated in food groups, we could not rule out the potential confounding influence of other nutrients. Additionally, we did not account for other factors related to the absorption of the mineral elements, such as the repression effect of calcium supplementation on iron absorption [66]. Finally, our study was based on the US cohort, and the generalizability of the conclusions was to be tested.

## 5. Conclusions

This cross-sectional study revealed that among middle-aged and older adults, a higher intake of dietary iron and copper was associated with worse cognitive function. Zinc and manganese intake were not significantly associated with CF. Further studies are needed to confirm the study findings and explore the underlying biological pathways.

## Figures and Tables

**Table 1:** Characteristics of the study participants in the Health and Retirement Study in 2014.

Characteristics	Overall (*n* = 6863)	Gender	
		Male (*n* = 2794)	Female (*n* = 4069)
**Age, years, mean (SD)**	66.7 (10.5)	66.8 (10.2)	66.7 (10.7)
**Race, *n* (%)**			
White	4783 (69.7)	1993 (71.3)	2790 (68.6)
Black	1098 (16.0)	394 (14.1)	704 (17.3)
Hispanic	765 (11.1)	320 (11.5)	445 (10.9)
Others	217 (3.2)	87 (3.1)	130 (3.2)
**Married or partnered, *n* (%)**	4417 (64.4)	2191 (78.4)	2226 (54.7)
**Education, years, mean (SD)**	13.0 (2.9)	13.2 (3.1)	12.9 (2.8)
**Total energy intake, kcal, mean (SD)**	1755.2 (682.3)	1876.0 (730.9)	1672.2 (633.8)
**Frequency of vigorous activity, *n* (%)**			
<1/month	3674 (53.5)	1250 (44.7)	2424 (59.6)
1–4/month	1448 (21.1)	701 (25.1)	747 (18.4)
>1/week	1741 (25.4)	843 (30.2)	898 (22.1)
**Household income, *n* (%)**			
0–<20,000	1508 (22.0)	439 (15.7)	1069 (26.3)
20,000–<40,000	1595 (23.2)	622 (22.3)	973 (23.9)
40,000–<80,000	1893 (27.6)	830 (29.7)	1063 (26.1)
≥80,000	1867 (27.2)	903 (32.3)	964 (23.7)
**Smoking status, *n* (%)**			
Never	3133 (45.7)	972 (34.8)	2161 (53.1)
Former	2989 (43.6)	1493 (53.4)	1496 (36.8)
Current	741 (10.8)	329 (11.8)	412 (10.1)
**Drinking status, *n* (%)**			
Never	3104 (45.2)	1043 (37.3)	2061 (50.7)
Former	1073 (15.6)	404 (14.5)	669 (16.4)
Current	2686 (39.1)	1347 (48.2)	1339 (32.9)
**Body weight status, *n* (%)**			
Underweight	63 (0.9)	13 (0.5)	50 (1.2)
Normal weight	1407 (20.5)	467 (16.7)	940 (23.1)
Overweight	2316 (33.7)	1084 (38.8)	1232 (30.3)
Obesity	3077 (44.8)	1230 (44.0)	1847 (45.4)
**Hypertension, *n* (%)**	4178 (60.9)	1739 (62.2)	2439 (59.9)
**Diabetes mellitus, *n* (%)**	1630 (23.8)	708 (25.3)	922 (22.7)
**Heart diseases, *n* (%)**	1724 (25.1)	843 (30.2)	881 (21.7)
**Depression, *n* (%)**	936 (13.6)	314 (11.2)	622 (15.3)
**Global cognitive score, mean (SD)**	15.3 (4.4)	15.0 (4.2)	15.5 (4.5)
**Dietary Iron intake, mg, mean (SD)**	13.3 (6.3)	14.1 (6.4)	12.8 (6.2)
**Dietary Copper intake, mg, mean (SD)**	1.4 (0.7)	1.4 (0.8)	1.3 (0.7)
**Dietary Zinc intake, mg, mean (SD)**	10.7 (4.6)	11.4 (4.9)	10.3 (4.4)
**Dietary Manganese intake, mg, mean (SD)**	3.3 (1.6)	3.3 (1.6)	3.2 (1.6)
**Vitamin supplement use, *n* (%)**	4578 (66.7)	1640 (58.7)	2938 (72.2)
**Iron supplement intake, *n* (%)**	1960 (28.6)	752 (26.9)	1208 (29.7)
**Zinc supplement intake, *n* (%)**	756 (11.0)	260 (9.3)	496 (12.2)

**Table 2:** Beta and 95% confidence intervals of global cognitive score according to dietary intake levels of iron, copper, zinc, and manganese.

	Range	Model 1	*p*-Value	Model 2	*p*-Value	Model 3	*p*-Value
**Iron**							
**Quintile 1**	**<8.1**	0.00 [Reference]		0.00 [Reference]		0.00 [Reference]	
**Quintile 2**	**8.1–<10.8**	**0.72 [0.41, 1.04]**	**<0.001**	**0.37 [0.08, 0.66]**	**0.012**	**0.30 [0.02, 0.59]**	**0.038**
**Quintile 3**	**10.8–<13.7**	**0.64 [0.30, 0.97]**	**<0.001**	0.13 [−0.18, 0.44]	0.411	0.01 [−0.30, 0.33]	0.941
**Quintile 4**	**13.7–<17.7**	**0.78 [0.40, 1.15]**	**<0.001**	0.20 [−0.15, 0.54]	0.264	0.05 [−0.31, 0.40]	0.794
**Quintile 5**	**≥17.7**	0.31 [−0.14, 0.76]	0.178	−0.26 [−0.68, 0.16]	0.221	**−0.50 [−0.94, −0.06]**	**0.027**
**P-trend**			0.847		0.116		**0.007**
**Copper**							
**Quintile 1**	**<0.8**	0.00 [Reference]		0.00 [Reference]		0.00 [Reference]	
**Quintile 2**	**0.8–<1.1**	**0.30 [−0.01, 0.61]**	**0.061**	−0.05 [−0.33, 0.24]	0.745	−0.11 [−0.39, 0.18]	0.461
**Quintile 3**	**1.1–<1.4**	**0.44 [0.12, 0.77]**	**0.008**	−0.03 [−0.33, 0.27]	0.832	−0.17 [−0.47, 0.14]	0.285
**Quintile 4**	**1.4–<1.8**	**0.46 [0.09, 0.83]**	**0.014**	−0.06 [−0.40, 0.28]	0.726	−0.27 [−0.63, 0.08]	0.124
**Quintile 5**	**≥1.8**	0.34 [−0.09, 0.77]	0.117	−0.19 [−0.59, 0.20]	0.344	**−0.52 [−0.94, −0.10]**	**0.014**
**P-trend**			0.176		0.064		**0.002**
**Zinc**							
**Quintile 1**	**<6.8**	0.00 [Reference]		0.00 [Reference]		0.00 [Reference]	
**Quintile 2**	**6.8–<9.0**	**0.50 [0.18, 0.81]**	**0.002**	0.15 [−0.14, 0.44]	0.314	0.10 [−0.19, 0.39]	0.494
**Quintile 3**	**9.0–<11.1**	**0.86 [0.52, 1.21]**	**<0.001**	0.26 [−0.06, 0.57]	0.110	0.18 [−0.14, 0.50]	0.266
**Quintile 4**	**11.1–<14.2**	**0.99 [0.60, 1.37]**	**<0.001**	0.28 [−0.08, 0.64]	0.124	0.17 [−0.19, 0.53]	0.348
**Quintile 5**	**≥14.2**	**1.05 [0.57, 1.53]**	**<0.001**	0.21 [−0.23, 0.66]	0.345	0.06 [−0.39, 0.51]	0.804
**P-trend**			**0.003**		0.553		0.785
**Manganese**							
**Quintile 1**	**<1.9**	0.00 [Reference]		0.00 [Reference]		0.00 [Reference]	
**Quintile 2**	**1.9–<2.6**	**0.75 [0.44, 1.06]**	**<0.001**	0.17 [−0.11, 0.46]	0.240	0.10 [−0.19, 0.39]	0.500
**Quintile 3**	**2.6–<3.4**	**1.04 [0.71, 1.36]**	**<0.001**	0.20 [−0.11, 0.50]	0.209	0.06 [−0.26, 0.38]	0.717
**Quintile 4**	**3.4–<4.4**	**1.51 [1.16, 1.86]**	**<0.001**	**0.48 [0.15, 0.81]**	**0.004**	0.26 [−0.10, 0.62]	0.161
**Quintile 5**	**≥4.4**	**1.68 [1.26, 2.09]**	**<0.001**	0.36 [−0.03, 0.75]	0.072	−0.03 [−0.50, 0.44]	0.912
**P-trend**			**<0.001**		**0.001**		0.368

Model 1 was adjusted for age, squared age, sex, and race. Model 2 was based on Model 1 and further adjusted for marriage status, education, physical activity, household income, smoking status, alcohol drinking status, and use of vitamin, iron, and zinc supplements. Model 3 was based on Model 2 and further adjusted for dietary intake levels of fat and fiber.

**Table 3:** Odds ratio and 95% confidence intervals of cognitive impairment according to dietary intake levels of iron, copper, zinc, and manganese.

	Range	Model 1	*p*-Value	Model 2	*p*-Value	Model 3	*p*-Value
**Iron**							
**Quintile 1**	**<8.1**	1.00 [Reference]		1.00 [Reference]		1.00 [Reference]	
**Quintile 2**	**8.1–<10.8**	**0.69 [0.56, 0.86]**	**0.001**	**0.79 [0.63, 0.99]**	**0.043**	0.82 [0.65, 1.02]	0.079
**Quintile 3**	**10.8–<13.7**	**0.77 [0.62, 0.96]**	**0.022**	0.96 [0.76, 1.22]	0.764	1.02 [0.80, 1.30]	0.866
**Quintile 4**	**13.7–<17.7**	0.80 [0.63, 1.03]	0.085	1.03 [0.79, 1.34]	0.825	1.11 [0.85, 1.45]	0.462
**Quintile 5**	**≥17.7**	0.97 [0.73, 1.31]	0.861	1.26 [0.92, 1.71]	0.151	**1.41 [1.02, 1.95]**	**0.040**
**P-trend**			0.197		0.059		**0.009**
**Copper**							
**Quintile 1**	**<0.8**	1.00 [Reference]		1.00 [Reference]		1.00 [Reference]	
**Quintile 2**	**0.8–<1.1**	0.96 [0.78, 1.18]	0.707	1.12 [0.90, 1.39]	0.320	1.14 [0.92, 1.43]	0.234
**Quintile 3**	**1.1–<1.4**	0.91 [0.73, 1.14]	0.407	1.11 [0.88, 1.40]	0.367	1.18 [0.93, 1.49]	0.177
**Quintile 4**	**1.4–<1.8**	0.88 [0.69, 1.13]	0.331	1.11 [0.86, 1.44]	0.425	1.21 [0.93, 1.58]	0.157
**Quintile 5**	**≥1.8**	1.08 [0.81, 1.43]	0.606	**1.35 [1.00, 1.81]**	**0.048**	**1.54 [1.13, 2.10]**	**0.006**
**P-trend**			0.052		0.062		**0.018**
**Zinc**							
**Quintile 1**	**<6.8**	1.00 [Reference]		1.00 [Reference]		1.00 [Reference]	
**Quintile 2**	**6.8–<9.0**	**0.77 [0.62, 0.95]**	**0.014**	0.88 [0.70, 1.09]	0.242	0.90 [0.72, 1.12]	0.332
**Quintile 3**	**9.0–<11.1**	**0.75 [0.60, 0.93]**	**0.011**	0.99 [0.78, 1.26]	0.962	1.03 [0.81, 1.31]	0.806
**Quintile 4**	**11.1–<14.2**	**0.63 [0.49, 0.81]**	**<0.001**	0.86 [0.66, 1.13]	0.290	0.91 [0.69, 1.19]	0.489
**Quintile 5**	**≥14.2**	**0.59 [0.43, 0.81]**	**0.001**	0.85 [0.60, 1.20]	0.354	0.92 [0.65, 1.29]	0.625
**P-trend**			0.057		0.887		0.668
**Manganese**							
**Quintile 1**	**<1.9**	1.00 [Reference]		1.00 [Reference]		1.00 [Reference]	
**Quintile 2**	**1.9–<2.6**	**0.62 [0.51, 0.76]**	**<0.001**	**0.79 [0.63, 0.98]**	**0.033**	0.82 [0.66, 1.02]	0.078
**Quintile 3**	**2.6–<3.4**	**0.66 [0.53, 0.82]**	**<0.001**	0.96 [0.76, 1.21]	0.720	1.03 [0.81, 1.31]	0.831
**Quintile 4**	**3.4–<4.4**	**0.50 [0.39, 0.63]**	**<0.001**	0.79 [0.61, 1.02]	0.071	0.88 [0.67, 1.16]	0.379
**Quintile 5**	**≥4.4**	**0.49 [0.37, 0.64]**	**<0.001**	0.87 [0.65, 1.18]	0.379	1.06 [0.74, 1.53]	0.743
**P-trend**			**<0.001**		0.187		0.944

Model 1 was adjusted for age, squared age, sex, and race. Model 2 was based on Model 1 and further adjusted for marriage status, education, physical activity, household income, smoking status, alcohol drinking status, and use of vitamin, iron, and zinc supplements. Model 3 was based on Model 2 and further adjusted for dietary intake levels of fat and fiber.

## Data Availability

The data used in this study are available in: https://hrsdata.isr.umich.edu/ (accessed on 20 May 2022).

## Supplementary Materials

The following are available online at https://www.mdpi.com/article/10.3390/nu15030704/s1, Table S1: Nutrient intake of participants calculated to total energy of 2500 kcal per day overall and by sex, Table S2: Subgroup associations of mineral intake with cognitive function, Table S3: Sensitivity analysis for further adjusted associations of mineral intake with cognitive function, Table S4: Sensitivity analysis for population excluded, mutual adjusted, and energy standardized associations of mineral intake with cognitive function, Table S5: Spearman’s rank correlation coefficient of dietary iron, copper, zinc, and manganese intake, Figure S1: Flow chart of the study population; Figure S2. Restricted cubic splines for the association of dietary intake of iron, copper, zinc, and manganese with cognitive impairment. Figure S3: Restricted cubic splines for the association of dietary intake of iron, copper, zinc, and manganese with global cognitive score by BMI, education, marital status, iron supplement intake, and zinc supplement intake.

## Abbreviations

Health and Retirement Study (HRS), Health Care and Nutrition Study (HCNS), standard deviation (SD), odds ratio (OR), confidence interval (CI), cognitive function (CF), cognitive impairment (CI).
