# Supplementary material for: Dietary Intake Levels of Iron, Copper, Zinc, and Manganese in Relation to Cognitive Function: A Cross-Sectional Study
Source: Nutrients. 2023 Jan 30;15(3):704. doi: 10.3390/nu15030704 (PMC9921562; doi:10.3390/nu15030704)
Supplement: Supplementary file 1 [file nutrients-15-00704-s001.zip › nutrients-2137309-supplementary.pdf]

Table S1. Spearman's rank correlation coefficient of dietary iron, copper, zinc, and manganese intake

Table S2. Nutrient intake of participants calculated to total energy of 2500 kcal per day overall and by sex

Table S3. Subgroup associations of mineral intake with cognitive function

Table S4. Sensitivity analysis for further adjusted associations of mineral intake with cognitive function

Table S5. Sensitivity analysis for population excluded, mutual adjusted, and energy standardized associations of mineral intake with cognitive function

Figure S1. Flow chart of the study population

Figure S2. Restricted cubic splines for the association of dietary intake of iron, copper, zinc, and manganese with cognitive impairment

Figure S3. Restricted cubic splines for the association of dietary intake of iron, copper, zinc, and manganese with global cognitive score by BMI, education, marital status, iron supplement intake, and zinc supplement intake

Table S1. Spearman's rank correlation coefficient of dietary iron, copper, zinc, and manganese intake

	Iron		Copper		Zinc	
	r	*P*-value	r	*P*-value	r	*P*-value
Copper	0.271	<0.001				
Zinc	0.569	<0.001	0.153	<0.001		
Manganese	0.535	<0.001	0.387	<0.001	0.299	<0.001

Table S2. Nutrient intake of participants calculated to total energy of 2500 kcal per day overall and by sex

Nutrients	Overall (n = 6863)	Gender	
		Male (n = 2794)	Female (n = 4069)
**Animal protein, g, mean (SD)**	63.52 (20.61)	62.17 (20.18)	64.45 (20.85)
**Vegetable protein, g, mean (SD)**	35.62 (11.87)	35.06 (10.45)	36.00 (12.75)
**Total sugar, g, mean (SD)**	144.90 (46.98)	140.29 (46.50)	148.07 (47.04)
**Dietary Fiber, g, mean (SD)**	27.21 (8.64)	25.79 (8.12)	28.18 (8.84)
**Other carbohydrates, g, mean (SD)**	143.15 (32.76)	145.12 (32.64)	141.79 (32.78)
**Saturated fat, g, mean (SD)**	31.05 (7.14)	30.88 (7.03)	31.17 (7.22)
**Monounsaturated fat, g, mean (SD)**	32.99 (7.63)	33.03 (7.22)	32.97 (7.90)
**Polyunsaturated fat, g, mean (SD)**	18.54 (5.65)	18.34 (5.37)	18.68 (5.82)
**Cholesterol, mg, mean (SD)**	338.13 (155.33)	348.47 (161.91)	331.03 (150.25)
**Iron, mg, mean (SD)**	19.20 (5.88)	19.15 (5.99)	19.23 (5.80)
**Magnesium, mg, mean (SD)**	415.15 (100.95)	402.77 (93.91)	423.65 (104.69)
**Phosphorus, mg, mean (SD)**	1,664.97 (337.65)	1,620.63 (309.86)	1,695.41 (352.30)
**Kalium, mg, mean (SD)**	3,983.19 (883.33)	3,816.70 (840.88)	4,097.52 (893.65)
**Sodium, mg, mean (SD)**	3,007.70 (714.59)	3,027.00 (734.18)	2,994.45 (700.60)
**Zinc, mg, mean (SD)**	15.45 (3.75)	15.38 (3.79)	15.50 (3.73)
**Copper, mg, mean (SD)**	1.97 (0.75)	1.91 (0.73)	2.01 (0.76)
**Manganese, mg, mean (SD)**	4.68 (1.52)	4.51 (1.51)	4.79 (1.52)
**Calcium, mg, mean (SD)**	1,043.72 (506.24)	974.60 (432.13)	1,091.18 (546.40)
**Alcohol, g, mean (SD)**	8.22 (16.63)	11.72 (20.08)	5.83 (13.24)
**Caffeine, mg, mean (SD)**	228.18 (227.21)	228.31 (213.67)	228.08 (236.08)
**Flavonoids, mg, mean (SD)**	414.95 (382.90)	374.22 (350.69)	442.93 (401.19)
**Vitamin C, mg, mean (SD)**	151.13 (88.25)	142.65 (86.52)	156.95 (88.95)
**Vitamin B1, mg, mean (SD)**	1.91 (1.31)	1.86 (1.01)	1.95 (1.48)

**Vitamin B2, mg, mean (SD)**	2.95 (1.49)	2.86 (1.22)	3.01 (1.65)
**Niacin, mg, mean (SD)**	29.03 (8.74)	28.96 (7.60)	29.08 (9.45)
**Pantothenic acid, mg, mean (SD)**	7.74 (2.54)	7.52 (2.36)	7.89 (2.64)
**Vitamin B6, mg, mean (SD)**	2.58 (0.72)	2.55 (0.69)	2.60 (0.74)
**Food folate, µg, mean (SD)**	379.73 (126.84)	360.36 (113.76)	393.02 (133.49)
**Vitamin B12, µg, mean (SD)**	9.13 (5.04)	9.08 (4.90)	9.17 (5.14)
**Retinol, µg, mean (SD)**	1,123.63 (548.47)	1,035.36 (503.65)	1,184.24 (569.43)
**Alpha carotene, µg, mean (SD)**	884.60 (899.18)	763.67 (751.28)	967.64 (979.48)
**Beta carotene, µg, mean (SD)**	4,945.97 (3,819.59)	4,107.73 (3,081.08)	5,521.54 (4,156.66)
**Beta cryptoxanthin, µg, mean (SD)**	183.75 (167.64)	180.85 (169.48)	185.75 (166.35)
**Lycopene, µg, mean (SD)**	7,052.27 (6,261.14)	7,173.85 (6,803.11)	6,968.79 (5,859.45)

Table S3. Subgroup associations of mineral intake with cognitive function

	Quintiles of mineral intake, mg									P-interaction
	Quintile 1	Quintile 2		Quintile 3		Quintile 4		Quintile 5		
		β (95% CI)	*P*-value	β (95% CI)	*P*-value	β (95% CI)	*P*-value	β (95% CI)	*P*-value	
**Iron**										
**Gender**										**0.049**
Male	Reference	0.43 [-0.05, 0.90]	0.078	0.19 [-0.31, 0.68]	0.458	0.31 [-0.23, 0.86]	0.261	-0.25 [-0.92, 0.42]	0.462	
Female	Reference	0.29 [-0.07, 0.66]	0.115	-0.02 [-0.43, 0.40]	0.931	-0.06 [-0.53, 0.42]	0.813	-0.54 [-1.14, 0.06]	0.078	
**Age, years**										0.051
<65	Reference	0.29 [-0.11, 0.69]	0.149	0.19 [-0.26, 0.65]	0.399	0.31 [-0.21, 0.82]	0.242	-0.14 [-0.78, 0.50]	0.676	
>=65	Reference	0.37 [-0.05, 0.79]	0.080	-0.15 [-0.59, 0.29]	0.513	-0.19 [-0.68, 0.30]	0.452	**-0.81 [-1.42, -0.19]**	**0.010**	
**Body mass index, kg/m^2**										0.900
< 25	Reference	0.43 [-0.23, 1.09]	0.202	0.15 [-0.54, 0.85]	0.670	0.15 [-0.62, 0.92]	0.702	-0.52 [-1.44, 0.41]	0.272	
>= 25	Reference	0.28 [-0.04, 0.60]	0.086	-0.01 [-0.37, 0.34]	0.938	0.06 [-0.34, 0.47]	0.753	-0.43 [-0.93, 0.07]	0.094	
**Education, years**										0.369
< 10	Reference	-0.39 [-1.48, 0.71]	0.490	-0.35 [-1.54, 0.83]	0.559	**-1.39 [-2.66, -0.11]**	**0.033**	**-1.61 [-3.15, -0.07]**	**0.040**	
10-15	Reference	**0.46 [0.10, 0.82]**	**0.012**	0.06 [-0.34, 0.46]	0.771	0.30 [-0.15, 0.75]	0.189	-0.21 [-0.78, 0.36]	0.470	
>= 15	Reference	0.15 [-0.37, 0.68]	0.573	0.03 [-0.54, 0.60]	0.925	0.03 [-0.61, 0.66]	0.937	-0.70 [-1.48, 0.09]	0.081	
**Marital status**										0.235
Married	Reference	0.31 [-0.05, 0.66]	0.090	-0.11 [-0.49, 0.28]	0.593	0.16 [-0.28, 0.59]	0.477	-0.27 [-0.81, 0.27]	0.329	
Unmarried	Reference	0.33 [-0.16, 0.82]	0.183	0.23 [-0.31, 0.77]	0.402	-0.14 [-0.76, 0.48]	0.662	**-0.88 [-1.64, -0.12]**	**0.024**	
**Cardiometabolic**										0.581
Yes	Reference	0.29 [-0.05, 0.64]	0.094	-0.05 [-0.43, 0.33]	0.784	0.02 [-0.41, 0.45]	0.930	**-0.54 [-1.08, -0.01]**	**0.045**	
No	Reference	0.35 [-0.17, 0.87]	0.187	0.17 [-0.40, 0.74]	0.557	0.14 [-0.48, 0.76]	0.654	-0.32 [-1.10, 0.46]	0.428	

**Iron supplement intake**									**0.016**
Yes	Reference	0.26 [-0.08, 0.59]	0.134	-0.06 [-0.43, 0.31]	0.761	-0.01 [-0.43, 0.41]	0.958	**-0.52 [-1.03, -0.00]**	**0.049**	
No	Reference	0.39 [-0.17, 0.95]	0.174	0.14 [-0.47, 0.75]	0.654	0.19 [-0.49, 0.87]	0.585	-0.38 [-1.25, 0.49]	0.395	
**Zinc supplement intake**										**0.027**
Yes	Reference	**0.32 [0.02, 0.62]**	**0.036**	0.01 [-0.32, 0.34]	0.962	0.03 [-0.35, 0.40]	0.892	-0.44 [-0.91, 0.02]	0.060	
No	Reference	0.06 [-0.92, 1.03]	0.912	-0.03 [-1.07, 1.02]	0.958	0.10 [-1.06, 1.27]	0.864	-0.89 [-2.36, 0.57]	0.232	
**Copper**										
**Gender**										**< 0.001**
Male	Reference	0.09 [-0.36, 0.54]	0.707	-0.00 [-0.48, 0.48]	0.999	0.48 [-0.05, 1.00]	0.076	0.26 [-0.37, 0.90]	0.416	
Female	Reference	-0.21 [-0.58, 0.16]	0.265	-0.25 [-0.64, 0.15]	0.223	**-0.80 [-1.27, -0.33]**	**0.001**	**-1.00 [-1.56, -0.45]**	**<0.001**	
**Age, years**										0.334
<65	Reference	-0.03 [-0.43, 0.37]	0.886	-0.21 [-0.64, 0.23]	0.349	-0.19 [-0.69, 0.31]	0.454	**-0.68 [-1.26, -0.09]**	**0.023**	
>=65	Reference	-0.15 [-0.55, 0.26]	0.474	-0.11 [-0.54, 0.32]	0.607	-0.30 [-0.80, 0.19]	0.229	-0.34 [-0.93, 0.26]	0.270	
**Body mass index, kg/m^2**										**0.040**
< 25	Reference	-0.04 [-0.70, 0.61]	0.898	0.28 [-0.41, 0.96]	0.431	-0.17 [-0.97, 0.62]	0.668	-0.57 [-1.47, 0.33]	0.214	
>= 25	Reference	-0.12 [-0.43, 0.20]	0.465	-0.28 [-0.62, 0.06]	0.105	-0.30 [-0.69, 0.10]	0.139	**-0.47 [-0.95, -0.00]**	**0.049**	
**Education, years**										0.169
< 10	Reference	0.09 [-1.00, 1.18]	0.871	-0.49 [-1.70, 0.72]	0.424	-0.74 [-2.12, 0.64]	0.292	-0.59 [-2.40, 1.21]	0.518	
10-15	Reference	0.04 [-0.31, 0.40]	0.805	-0.06 [-0.46, 0.34]	0.774	0.20 [-0.26, 0.65]	0.394	-0.13 [-0.73, 0.48]	0.685	
>= 15	Reference	0.20 [-0.36, 0.76]	0.486	0.43 [-0.16, 1.01]	0.155	**0.69 [0.04, 1.35]**	**0.038**	0.50 [-0.32, 1.32]	0.231	
**Marital status**										0.240
Married	Reference	-0.01 [-0.36, 0.34]	0.953	-0.22 [-0.59, 0.16]	0.253	0.04 [-0.40, 0.47]	0.871	-0.19 [-0.71, 0.34]	0.488	
Unmarried	Reference	-0.22 [-0.71, 0.27]	0.384	0.04 [-0.49, 0.57]	0.883	**-0.75 [-1.35, -0.15]**	**0.014**	**-0.97 [-1.66, -0.29]**	**0.006**	
**Cardiometabolic**										0.338
Yes	Reference	-0.17 [-0.51, 0.17]	0.323	-0.23 [-0.60, 0.13]	0.214	-0.40 [-0.82, 0.02]	0.060	**-0.58 [-1.08, -0.09]**	**0.021**	

No	Reference	0.05 [-0.47, 0.57]	0.857	0.05 [-0.50, 0.61]	0.851	0.08 [-0.57, 0.73]	0.814	-0.22 [-1.00, 0.56]	0.578	
**Iron supplement intake**										0.095
Yes	Reference	-0.21 [-0.54, 0.13]	0.224	-0.25 [-0.61, 0.10]	0.164	-0.41 [-0.83, 0.00]	0.052	**-0.62 [-1.11, -0.14]**	**0.011**	
No	Reference	0.11 [-0.45, 0.68]	0.693	0.03 [-0.55, 0.62]	0.910	0.05 [-0.62, 0.71]	0.894	-0.19 [-1.02, 0.65]	0.659	
**Zinc supplement intake**										0.141
Yes	Reference	-0.10 [-0.40, 0.20]	0.514	-0.18 [-0.49, 0.14]	0.280	-0.27 [-0.64, 0.10]	0.155	**-0.49 [-0.93, -0.05]**	**0.028**	
No	Reference	-0.20 [-1.22, 0.83]	0.707	-0.07 [-1.13, 0.99]	0.898	-0.42 [-1.58, 0.73]	0.474	-0.70 [-2.05, 0.65]	0.306	
**Zinc**										
**Gender**										0.285
Male	Reference	0.02 [-0.45, 0.50]	0.930	0.24 [-0.27, 0.74]	0.356	0.27 [-0.28, 0.82]	0.335	-0.06 [-0.75, 0.62]	0.855	
Female	Reference	0.20 [-0.17, 0.57]	0.288	0.25 [-0.17, 0.66]	0.244	0.19 [-0.29, 0.67]	0.445	0.30 [-0.30, 0.91]	0.328	
**Age, years**										**0.011**
<65	Reference	0.26 [-0.15, 0.68]	0.212	0.36 [-0.09, 0.82]	0.118	0.44 [-0.07, 0.95]	0.092	0.56 [-0.08, 1.20]	0.085	
>=65	Reference	-0.04 [-0.44, 0.37]	0.855	0.04 [-0.41, 0.48]	0.868	-0.07 [-0.58, 0.43]	0.780	-0.47 [-1.10, 0.16]	0.145	
**Body mass index, kg/m^2**										0.306
< 25	Reference	0.38 [-0.26, 1.02]	0.244	0.48 [-0.21, 1.17]	0.172	0.67 [-0.11, 1.45]	0.093	0.49 [-0.49, 1.47]	0.329	
>= 25	Reference	0.03 [-0.29, 0.36]	0.849	0.10 [-0.26, 0.45]	0.596	0.04 [-0.36, 0.44]	0.847	-0.04 [-0.55, 0.47]	0.873	
**Education, years**										0.401
< 10	Reference	0.15 [-0.37, 0.68]	0.573	0.03 [-0.54, 0.60]	0.925	0.03 [-0.61, 0.66]	0.937	-0.70 [-1.48, 0.09]	0.081	
10-15	Reference	-0.17 [-1.22, 0.88]	0.750	-0.16 [-1.35, 1.03]	0.791	-0.68 [-2.01, 0.65]	0.317	-0.89 [-2.47, 0.70]	0.273	
>= 15	Reference	0.11 [-0.25, 0.48]	0.539	0.15 [-0.25, 0.56]	0.467	0.32 [-0.14, 0.78]	0.174	0.36 [-0.22, 0.95]	0.226	
**Marital status**										0.242
Married	Reference	0.22 [-0.14, 0.58]	0.228	0.15 [-0.24, 0.54]	0.450	0.38 [-0.06, 0.81]	0.092	0.25 [-0.29, 0.80]	0.365	
Unmarried	Reference	-0.09 [-0.57, 0.40]	0.723	0.33 [-0.22, 0.87]	0.239	-0.18 [-0.80, 0.44]	0.569	-0.30 [-1.10, 0.49]	0.454	
**Cardiometabolic**										0.243

Yes	Reference	0.02 [-0.33, 0.36]	0.923	-0.02 [-0.40, 0.36]	0.901	0.00 [-0.43, 0.44]	0.982	-0.20 [-0.74, 0.35]	0.477	
No	Reference	0.32 [-0.21, 0.85]	0.243	**0.64 [0.07, 1.22]**	**0.028**	0.58 [-0.05, 1.22]	0.071	0.74 [-0.06, 1.54]	0.070	
**Iron supplement intake**										**0.005**
Yes	Reference	0.10 [-0.23, 0.44]	0.544	0.18 [-0.19, 0.55]	0.342	0.25 [-0.17, 0.67]	0.248	0.19 [-0.34, 0.72]	0.476	
No	Reference	0.09 [-0.48, 0.66]	0.747	0.11 [-0.50, 0.72]	0.719	-0.03 [-0.72, 0.66]	0.933	-0.21 [-1.07, 0.64]	0.621	
**Zinc supplement intake**										**0.029**
Yes	Reference	0.12 [-0.18, 0.42]	0.445	0.15 [-0.19, 0.48]	0.385	0.14 [-0.24, 0.51]	0.473	0.08 [-0.39, 0.55]	0.738	
No	Reference	-0.07 [-1.05, 0.92]	0.893	0.43 [-0.63, 1.49]	0.427	0.46 [-0.77, 1.69]	0.465	-0.04 [-1.58, 1.51]	0.962	
**Manganese**										
**Gender**										0.133
Male	Reference	-0.17 [-0.62, 0.28]	0.466	-0.07 [-0.56, 0.42]	0.776	0.39 [-0.16, 0.94]	0.167	-0.12 [-0.83, 0.59]	0.736	
Female	Reference	0.29 [-0.08, 0.67]	0.128	0.18 [-0.25, 0.60]	0.415	0.23 [-0.25, 0.70]	0.353	0.09 [-0.55, 0.72]	0.785	
**Age, years**										0.188
<65	Reference	0.07 [-0.33, 0.48]	0.728	0.20 [-0.25, 0.66]	0.378	**0.57 [0.06, 1.07]**	**0.028**	0.24 [-0.41, 0.89]	0.466	
>=65	Reference	0.15 [-0.27, 0.57]	0.480	-0.07 [-0.52, 0.39]	0.770	-0.00 [-0.52, 0.52]	0.998	-0.27 [-0.95, 0.42]	0.450	
**Body mass index, kg/m^2**										**0.006**
< 25	Reference	**0.74 [0.06, 1.42]**	**0.033**	0.53 [-0.19, 1.26]	0.149	0.62 [-0.17, 1.42]	0.123	0.65 [-0.35, 1.66]	0.203	
>= 25	Reference	-0.05 [-0.37, 0.27]	0.750	-0.06 [-0.41, 0.30]	0.758	0.18 [-0.23, 0.58]	0.394	-0.22 [-0.75, 0.32]	0.429	
**Education, years**										0.523
< 10	Reference	0.11 [-0.25, 0.48]	0.539	0.15 [-0.25, 0.56]	0.467	0.32 [-0.14, 0.78]	0.174	0.36 [-0.22, 0.95]	0.226	
10-15	Reference	0.15 [-0.38, 0.68]	0.586	0.32 [-0.24, 0.88]	0.261	0.16 [-0.47, 0.79]	0.621	-0.20 [-0.98, 0.58]	0.613	
>= 15	Reference	0.09 [-1.00, 1.18]	0.871	-0.49 [-1.70, 0.72]	0.424	-0.74 [-2.12, 0.64]	0.292	-0.59 [-2.40, 1.21]	0.518	
**Marital status**										0.358
Married	Reference	0.10 [-0.25, 0.46]	0.568	0.18 [-0.21, 0.57]	0.374	**0.48 [0.04, 0.92]**	**0.031**	0.11 [-0.46, 0.68]	0.706	
Unmarried	Reference	0.09 [-0.41, 0.59]	0.721	-0.17 [-0.73, 0.38]	0.545	-0.18 [-0.81, 0.46]	0.584	-0.28 [-1.12, 0.56]	0.507	

**Cardiometabolic**										0.817
Yes	Reference	0.11 [-0.24, 0.45]	0.538	-0.00 [-0.39, 0.38]	0.991	0.25 [-0.19, 0.69]	0.270	0.08 [-0.50, 0.66]	0.789	
No	Reference	0.14 [-0.40, 0.67]	0.616	0.29 [-0.28, 0.87]	0.318	0.40 [-0.24, 1.03]	0.223	-0.05 [-0.86, 0.77]	0.912	
**Iron supplement intake**										0.087
Yes	Reference	0.10 [-0.23, 0.44]	0.551	0.11 [-0.26, 0.48]	0.565	0.22 [-0.21, 0.65]	0.315	0.13 [-0.43, 0.69]	0.657	
No	Reference	0.01 [-0.57, 0.60]	0.960	-0.17 [-0.80, 0.45]	0.583	0.22 [-0.47, 0.90]	0.534	-0.41 [-1.29, 0.48]	0.370	
**Zinc supplement intake**										0.274
Yes	Reference	0.07 [-0.24, 0.37]	0.663	0.08 [-0.25, 0.42]	0.623	0.26 [-0.12, 0.64]	0.185	-0.02 [-0.52, 0.48]	0.945	
No	Reference	0.32 [-0.71, 1.35]	0.542	-0.24 [-1.32, 0.83]	0.660	0.15 [-1.02, 1.32]	0.795	-0.15 [-1.64, 1.33]	0.839	

All subgroup analyses were based on Model 3, which adjusted for age, squared age, sex, race, marriage status, education, physical activity, household income, smoking status, alcohol drinking status, use of vitamin, iron, and zinc supplements, and dietary intake of fat and fiber.

BMI: body mass index

Table S4. Sensitivity analysis for further adjusted associations of mineral intake with cognitive function

	Range	Model 4	*P*-value	Model 5	*P*-value	Model 6	*P*-value	Model 7	*P*-value
**Iron**									
**Quintile 1**	**<8.1**	0.00 [Reference]		0.00 [Reference]		0.00 [Reference]		0.00 [Reference]	
**Quintile 2**	**8.1-<10.8**	**0.31 [0.02, 0.60]**	**0.036**	**0.33 [0.05, 0.62]**	**0.022**	**0.30 [0.01, 0.59]**	**0.042**	**0.29 [0.00, 0.58]**	**0.048**
**Quintile 3**	**10.8-<13.7**	0.02 [-0.30, 0.33]	0.912	0.03 [-0.28, 0.35]	0.849	0.01 [-0.31, 0.32]	0.956	-0.02 [-0.33, 0.30]	0.921
**Quintile 4**	**13.7-<17.7**	0.05 [-0.30, 0.40]	0.784	0.06 [-0.30, 0.41]	0.760	0.05 [-0.31, 0.40]	0.803	0.01 [-0.35, 0.36]	0.969
**Quintile 5**	**>=17.7**	**-0.46 [-0.90, -0.02]**	**0.039**	**-0.45 [-0.89, -0.01]**	**0.046**	**-0.51 [-0.95, -0.06]**	**0.025**	**-0.59 [-1.04, -0.15]**	**0.009**
**p-trend**			**0.012**		**0.012**		**0.006**		**0.001**
**Copper**									
**Quintile 1**	**<0.8**	0.00 [Reference]		0.00 [Reference]		0.00 [Reference]		0.00 [Reference]	
**Quintile 2**	**0.8-<1.1**	-0.11 [-0.39, 0.17]	0.447	-0.10 [-0.38, 0.19]	0.501	-0.11 [-0.39, 0.18]	0.466	-0.13 [-0.41, 0.16]	0.388
**Quintile 3**	**1.1-<1.4**	-0.16 [-0.46, 0.15]	0.316	-0.15 [-0.45, 0.15]	0.334	-0.17 [-0.47, 0.14]	0.280	-0.20 [-0.50, 0.11]	0.208
**Quintile 4**	**1.4-<1.8**	-0.25 [-0.60, 0.10]	0.161	-0.28 [-0.62, 0.07]	0.122	-0.28 [-0.63, 0.07]	0.119	-0.31 [-0.67, 0.04]	0.081
**Quintile 5**	**>=1.8**	**-0.48 [-0.90, -0.07]**	**0.023**	**-0.47 [-0.88, -0.05]**	**0.028**	**-0.52 [-0.94, -0.10]**	**0.015**	**-0.62 [-1.04, -0.19]**	**0.005**
**p-trend**			**0.004**		**0.005**		**0.002**		**<0.001**
**Zinc**									
**Quintile 1**	**<6.8**	0.00 [Reference]		0.00 [Reference]		0.00 [Reference]		0.00 [Reference]	
**Quintile 2**	**6.8-<9.0**	0.11 [-0.18, 0.40]	0.445	0.12 [-0.17, 0.40]	0.434	0.09 [-0.20, 0.38]	0.544	0.04 [-0.25, 0.33]	0.786
**Quintile 3**	**9.0-<11.1**	0.20 [-0.12, 0.51]	0.224	0.19 [-0.13, 0.50]	0.241	0.17 [-0.15, 0.49]	0.299	0.08 [-0.25, 0.40]	0.645
**Quintile 4**	**11.1-<14.2**	0.19 [-0.16, 0.55]	0.286	0.19 [-0.17, 0.55]	0.295	0.16 [-0.20, 0.51]	0.396	0.00 [-0.38, 0.38]	0.983
**Quintile 5**	**>=14.2**	0.11 [-0.34, 0.56]	0.617	0.08 [-0.36, 0.53]	0.710	0.03 [-0.42, 0.48]	0.910	-0.21 [-0.71, 0.28]	0.402
**p-trend**			0.961		0.948		0.680		0.096
**Manganese**									
**Quintile 1**	**<1.9**	0.00 [Reference]		0.00 [Reference]		0.00 [Reference]		0.00 [Reference]	
**Quintile 2**	**1.9-<2.6**	0.10 [-0.19, 0.39]	0.492	0.10 [-0.19, 0.39]	0.504	0.10 [-0.19, 0.39]	0.505	0.09 [-0.20, 0.38]	0.541
**Quintile 3**	**2.6-<3.4**	0.06 [-0.26, 0.38]	0.717	0.06 [-0.26, 0.38]	0.721	0.06 [-0.26, 0.38]	0.704	0.04 [-0.28, 0.36]	0.793

**Quintile 4**	**3.4-<4.4**	0.26 [-0.10, 0.62]	0.164	0.25 [-0.11, 0.61]	0.178	0.27 [-0.09, 0.63]	0.147	0.24 [-0.12, 0.60]	0.197
**Quintile 5**	**>=4.4**	-0.02 [-0.49, 0.45]	0.936	-0.01 [-0.48, 0.46]	0.961	-0.01 [-0.49, 0.46]	0.951	-0.09 [-0.56, 0.39]	0.715
**p-trend**			0.354		0.343		0.326		0.605

Model 4 was based on Model 3 and further adjusted for hypertension, diabetes mellitus, heart problems.

Model 5 was based on Model 3 and further adjusted for depression.

Model 6 was based on Model 3 and further adjusted for body mass index (BMI).

Model 7 was based on Model 3 and further adjusted for intake of pantothenic and phosphorous.

Table S5. Sensitivity analysis for population excluded, mutual adjusted, and energy standardized associations of mineral intake with cognitive function

	Range	Model 8	*P*-value	Model 9	*P*-value	Model 10	*P*-value	Model 11	*P*-value
**Iron**									
**Quintile 1**	**<8.1**	0.00 [Reference]		0.00 [Reference]		0.00 [Reference]		0.00 [Reference]	
**Quintile 2**	**8.1-<10.8**	0.25 [-0.03, 0.54]	0.076	0.27 [-0.03, 0.56]	0.077	0.25 [-0.04, 0.54]	0.092	0.27 [-0.01, 0.55]	0.058
**Quintile 3**	**10.8-<13.7**	0.12 [-0.20, 0.43]	0.467	0.03 [-0.29, 0.36]	0.837	-0.09 [-0.42, 0.24]	0.609	0.16 [-0.12, 0.45]	0.267
**Quintile 4**	**13.7-<17.7**	0.19 [-0.16, 0.54]	0.291	0.11 [-0.25, 0.48]	0.541	-0.11 [-0.49, 0.28]	0.591	-0.00 [-0.29, 0.28]	0.979
**Quintile 5**	**>=17.7**	-0.12 [-0.56, 0.31]	0.579	-0.34 [-0.80, 0.11]	0.138	**-0.75 [-1.26, -0.24]**	**0.004**	-0.14 [-0.44, 0.16]	0.355
**p-trend**			0.111		**0.033**		**< 0.001**		**0.006**
**Copper**									
**Quintile 1**	**<0.8**	0.00 [Reference]		0.00 [Reference]		0.00 [Reference]		0.00 [Reference]	
**Quintile 2**	**0.8-<1.1**	-0.04 [-0.32, 0.24]	0.761	-0.14 [-0.43, 0.15]	0.350	-0.13 [-0.42, 0.15]	0.363	-0.01 [-0.30, 0.27]	0.933
**Quintile 3**	**1.1-<1.4**	-0.16 [-0.45, 0.14]	0.306	-0.17 [-0.48, 0.15]	0.299	-0.20 [-0.50, 0.11]	0.211	0.06 [-0.23, 0.36]	0.674
**Quintile 4**	**1.4-<1.8**	-0.22 [-0.56, 0.13]	0.219	-0.31 [-0.67, 0.05]	0.094	-0.32 [-0.67, 0.03]	0.075	0.04 [-0.25, 0.34]	0.772
**Quintile 5**	**>=1.8**	-0.37 [-0.78, 0.04]	0.076	**-0.49 [-0.92, -0.06]**	**0.024**	**-0.58 [-1.00, -0.16]**	**0.007**	**-0.49 [-0.79, -0.19]**	**0.001**
**p-trend**			**0.026**		**0.007**		**0.002**		**<0.001**
**Zinc**									
**Quintile 1**	**<6.8**	0.00 [Reference]		0.00 [Reference]		0.00 [Reference]		0.00 [Reference]	
**Quintile 2**	**6.8-<9.0**	0.11 [-0.18, 0.39]	0.466	0.10 [-0.20, 0.40]	0.513	0.17 [-0.12, 0.46]	0.255	-0.21 [-0.49, 0.07]	0.137
**Quintile 3**	**9.0-<11.1**	0.24 [-0.07, 0.55]	0.135	0.13 [-0.20, 0.46]	0.434	0.31 [-0.02, 0.64]	0.066	0.05 [-0.23, 0.33]	0.722
**Quintile 4**	**11.1-<14.2**	0.28 [-0.08, 0.63]	0.125	0.19 [-0.18, 0.56]	0.324	0.37 [-0.02, 0.75]	0.060	-0.05 [-0.33, 0.23]	0.726
**Quintile 5**	**>=14.2**	0.24 [-0.21, 0.68]	0.296	0.17 [-0.29, 0.64]	0.462	0.44 [-0.07, 0.95]	0.088	0.08 [-0.20, 0.36]	0.570
**p-trend**			0.379		0.654		**0.033**		0.723
**Manganese**									
**Quintile 1**	**<1.9**	0.00 [Reference]		0.00 [Reference]		0.00 [Reference]		0.00 [Reference]	
**Quintile 2**	**1.9-<2.6**	0.01 [-0.28, 0.29]	0.958	0.07 [-0.23, 0.36]	0.667	0.14 [-0.16, 0.43]	0.361	-0.23 [-0.51, 0.06]	0.119

**Quintile 3**	**2.6-<3.4**	0.05 [-0.26, 0.36]	0.755	0.09 [-0.24, 0.41]	0.610	0.12 [-0.20, 0.45]	0.460	-0.04 [-0.34, 0.26]	0.810
**Quintile 4**	**3.4-<4.4**	0.24 [-0.12, 0.59]	0.191	0.30 [-0.07, 0.68]	0.110	0.35 [-0.02, 0.72]	0.065	-0.14 [-0.46, 0.18]	0.383
**Quintile 5**	**>=4.4**	-0.04 [-0.50, 0.43]	0.876	-0.02 [-0.51, 0.46]	0.932	0.16 [-0.33, 0.65]	0.521	0.04 [-0.32, 0.40]	0.837
**p-trend**			0.368		0.318		**0.026**		0.358

Model 8 was based on Model 3 and excluded participants with global cognitive score <= 6.

Model 9 was based on Model 3 and excluded participants with stroke history.

Model 10 was based on Model 3 and further adjusted for the other three mineral intake when treating certain mineral intake as exposure.

Model 11 was based on Model 3 and standardized dietary intake.

Figure S1. Flow chart of the study population

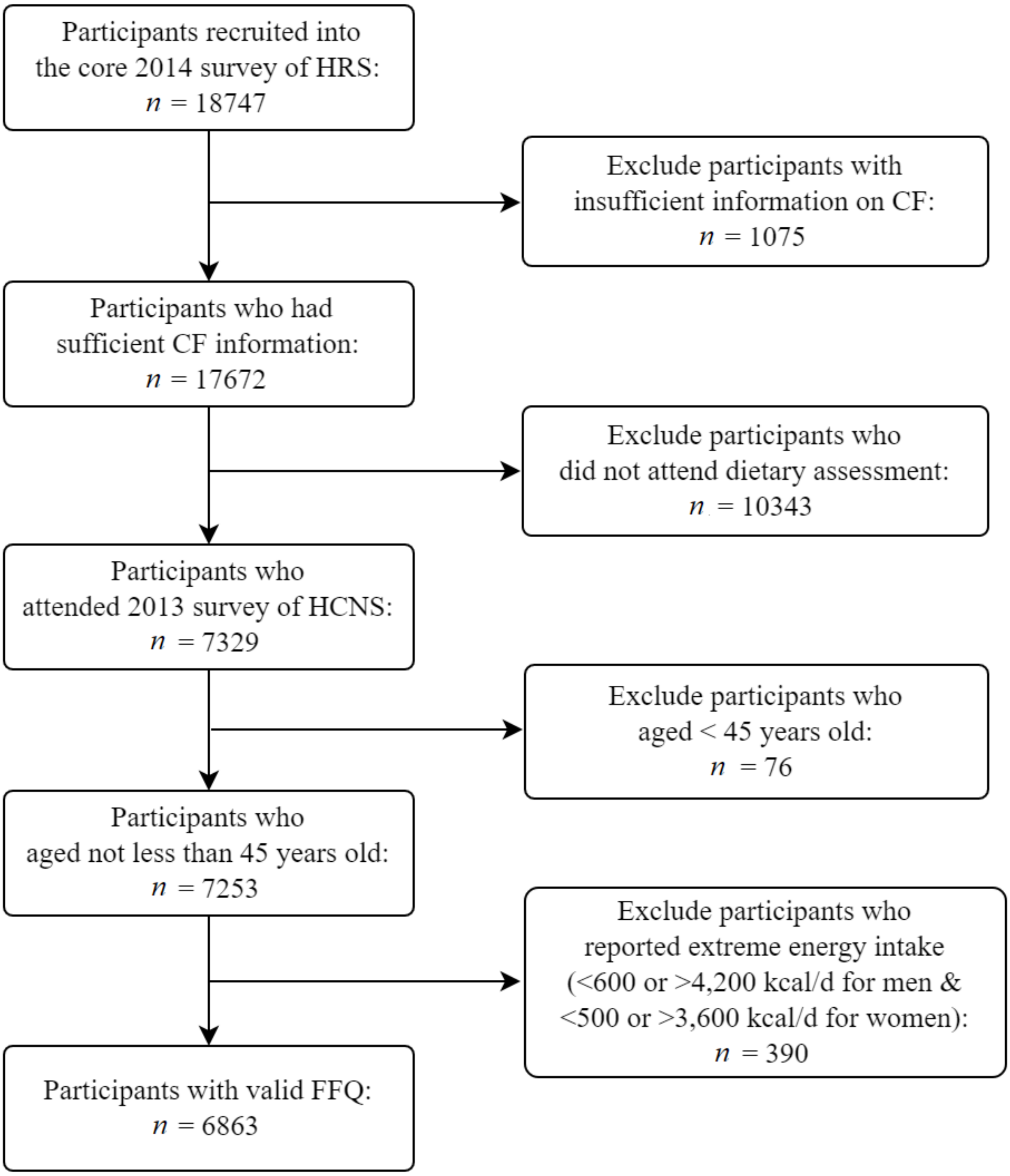

HRS: the Health and Retirement Study, CF: cognitive function, HCNS: the Health Care and Nutritional Study, FFQ: Food Frequency Questionnaires.

Figure S2. Restricted cubic splines for the association of dietary intake of iron, copper, zinc, and manganese with cognitive impairment

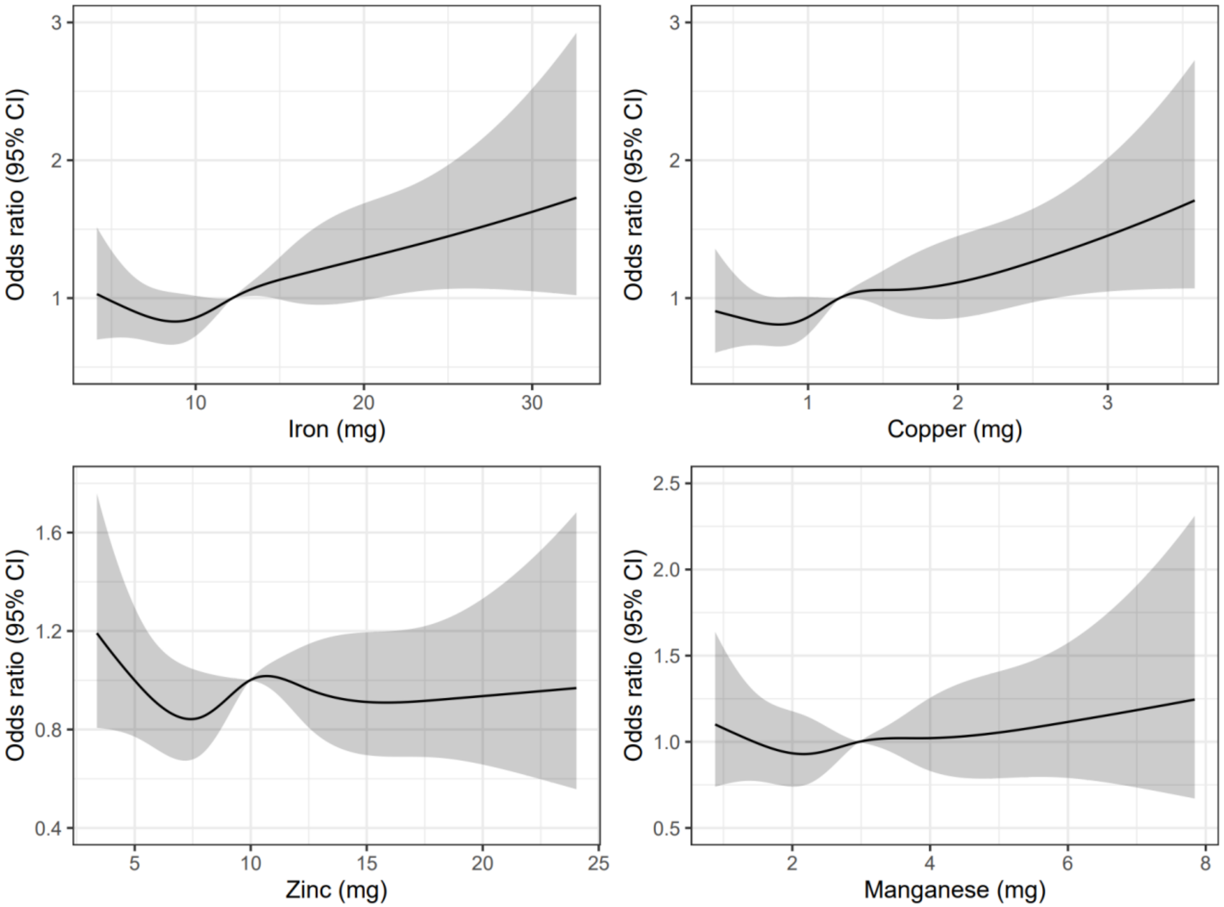

The reference point was set as the median values of dietary intake of iron, copper, zinc, and manganese. P-nonlinear = 0.350 for dietary iron intake, 0.634 for dietary copper intake, 0.307 for dietary zinc intake, and 0.786 for dietary manganese intake. The solid line represents the ORs of the logistic model, and the shaded area represents the 95% CIs of ORs.

Figure S3. Restricted cubic splines for the association of dietary intake of iron, copper, zinc, and manganese with global cognitive score by BMI, education, marital status, iron supplement intake, and zinc supplement intake

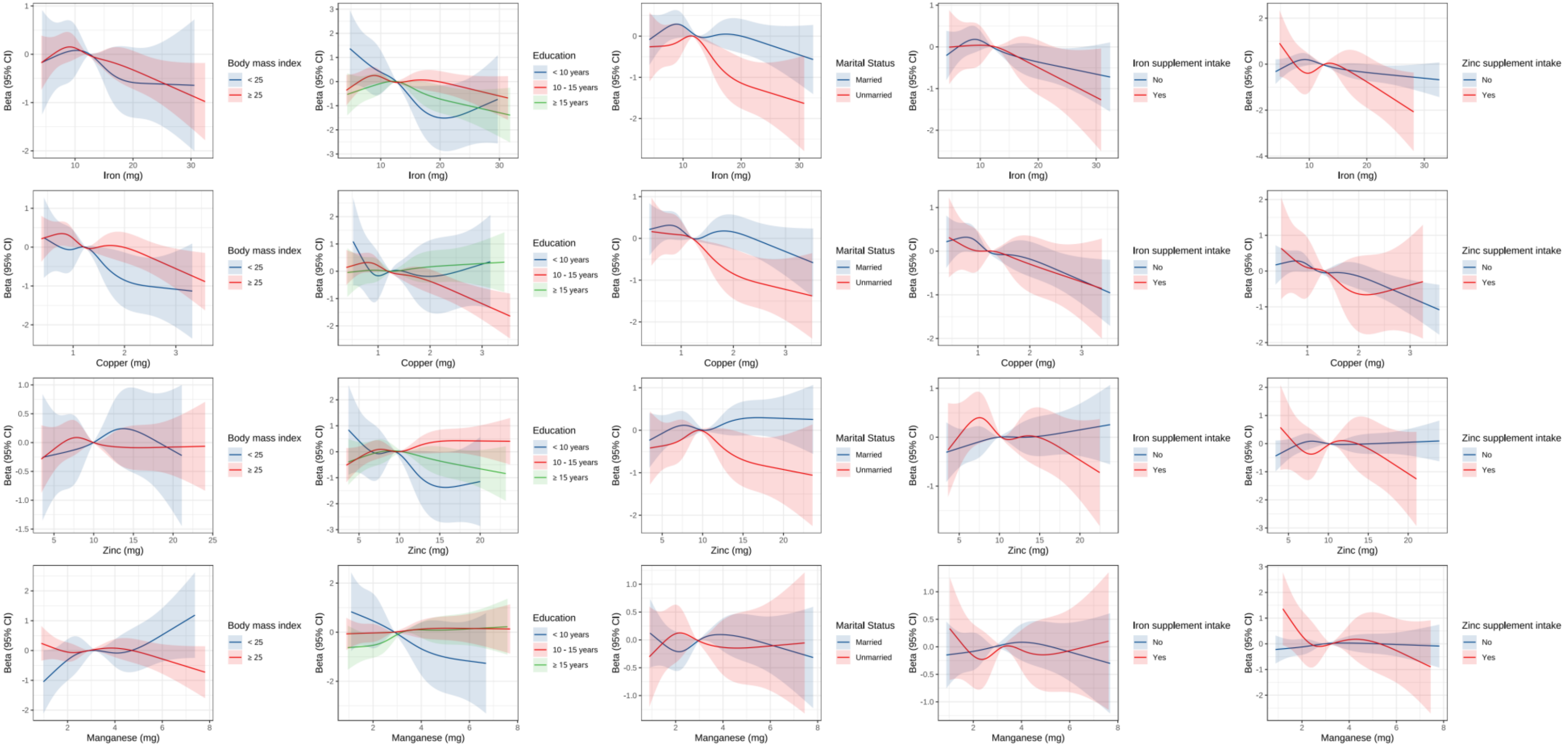

The solid line represents the beta of linear model, and shaded area represents the 95% CIs of beta
